# Visceral Adiposity Index in Coronary Artery Ectasia

**DOI:** 10.5152/eurasianjmed.2026.261588

**Published:** 2026-09-23

**Authors:** Vedat Aslan, Sefa Sural

**Affiliations:** Vocational School of Health Service, Toros University, Mersin, Türkiye

**Keywords:** Cardiometabolic dysfunction, coronary artery ectasia, propensity score matching, vascular remodeling, visceral adiposity index

## Abstract

**Background:**

Coronary artery ectasia (CAE) describes abnormal widening of the epicardial coronary arteries, generally attributed to dysregulated remodeling of the arterial wall. Combining body composition and lipid parameters, the visceral adiposity index (VAI) provides an indirect reflection of dysfunctional adipose tissue and related metabolic risk. The analysis specifically evaluated differences in VAI between patients with isolated CAE and those with unremarkable coronary angiograms, as well as the independent association between VAI and CAE.

**Methods:**

Records of adults who underwent coronary angiography at a single tertiary‑care institution between March 2024 and March 2026 were retrospectively reviewed. Isolated CAE was defined as ectatic dilatation of at least 1 epicardial coronary artery to ≥1.5 times the adjacent reference diameter, without stenosis of ≥50% in any major epicardial vessel. To reduce baseline imbalance, propensity score matching was applied, yielding 57 isolated-CAE patients and 57 patients with normal coronary arteries (NCA). Sex-specific formulas were used to derive each patient’s VAI. The relationship of VAI–CAE was modeled using multivariable logistic regression, and its discriminative capacity was characterized through receiver operating characteristic curve analysis.

**Results:**

The final matched sample consisted of 114 patients. Visceral adiposity index was higher among those with CAE than among NCA controls (3.01 ± 1.49 vs. 1.95 ± 1.05;*P* < .001), and the CAE group likewise showed greater body mass index, waist circumference, and triglycerides alongside lower high-density lipoprotein cholesterol (all *P* < .05). This VAI–CAE association persisted after multivariable adjustment.

**Conclusion:**

Isolated CAE was linked to a metabolic profile suggestive of unfavorable visceral adiposity. While VAI could add complementary metabolic insight in this patient group, larger prospective studies are needed before firmer conclusions can be drawn.

**Main Points**

- Following propensity score matching, patients with isolated coronary artery ectasia (CAE) continued to show significantly higher VAI values than those with normal coronary arteries.
- A 1-unit rise in Visceral Adiposity Index (VAI) corresponded to greater odds of CAE, an association that persisted after multivariable adjustment.
- In the matched cohort, VAI distinguished CAE from normal coronary arteries with moderate accuracy, yielding an area under the curve of 0.757.
- Overall, the results connect isolated CAE to a metabolic phenotype marked by visceral-adiposity–related dysfunction.
- Visceral adiposity index appears useful as a complementary tool for metabolic assessment in CAE, though it should not be used alone for diagnosis or risk stratification.

## Introduction

Even with considerable progress in preventive and therapeutic strategies, cardiovascular disease remains one of the leading contributors to global mortality and long-term disability. The extent of luminal narrowing alone does not fully explain coronary pathology, which instead arises from the interplay of metabolic, inflammatory, oxidative, and endothelial mechanisms. Together, abnormal lipid handling, inflammatory activity within the vessel wall, oxidative damage, and endothelial dysfunction shape both the onset and the course of coronary artery disease.^1^^,^^2^

On coronary angiography, coronary artery ectasia (CAE) is diagnosed when an arterial segment’s diameter is at least 1.5 times that of the adjacent normal-appearing vessel.^3^ It commonly overlaps with atherosclerotic coronary artery disease (CAD); yet, its structural and biological features point to a distinct, heterogeneous vascular process rather than a straightforward atherosclerotic subtype.^4^^-^^6^ Series based on coronary angiography estimate that CAE affects roughly 1%-5% of patients undergoing the procedure.^4^^-^^6^ Candidate mechanisms underlying its development include breakdown of the extracellular matrix, ongoing inflammatory activity, maladaptive remodeling of the arterial wall, and progressive structural weakening.^6^^,^^7^ Such changes can compromise coronary flow and have been linked to myocardial ischemia and adverse cardiac outcomes.^8^^-^^10^

Far from being an inert fat depot, visceral adipose tissue behaves as an endocrine organ, secreting adipokines, free fatty acids, and inflammatory mediators. Once this tissue becomes excessive or dysfunctional, it can drive insulin resistance, oxidative stress, endothelial damage, and chronic low-grade systemic inflammation,^11^^-^^13^ processes that together may promote vascular injury and abnormal remodeling of the cardiovascular system. Combining body mass index (BMI), waist circumference (WCF), triglyceride (TG) concentration, and high-density lipoprotein cholesterol (HDL-C) into a single index, the visceral adiposity index (VAI) has been put forward as a marker of adipose tissue dysfunction and associated cardiometabolic risk,^14^^,^^15^ one that may capture metabolic derangements more accurately than anthropometry alone. Elevated VAI has previously been linked to greater risk of cardiovascular disease, atherosclerotic disease, and cardiovascular death.^13^

While the literature linking visceral adiposity to obstructive CAD has grown considerably, its relevance to isolated CAE has received comparatively little attention.^13^^,^^16^^,^^17^ The present study was therefore designed to determine whether VAI values differ between patients with isolated CAE and angiographically normal controls and to quantify the strength of any association between VAI and this ectatic coronary phenotype.

## Material and Methods

### Ethical Statement

Approval for this study was granted by the institutional review board on April 1, 2026 (protocol no. 2026/21), and all study procedures were carried out in line with the Declaration of Helsinki. Because the analysis relied exclusively on anonymized, retrospectively collected clinical data, a waiver of individual informed consent applied. Reporting followed the Strengthening the Reporting of Observational Studies in Epidemiology checklist for observational studies.^18^

### Study Setting and Population

The institution’s coronary angiography registry for all procedures carried out between March 2024 and March 2026 was screened. Of 2120 angiographic studies, 102 met the diagnostic criterion for CAE, giving a prevalence of 4.81%. Of these, 32 patients were later excluded from the analysis for the following reasons: acute coronary syndrome (n = 9), valvular heart disease (n = 4), active infection or inflammation (n = 3), missing clinical or laboratory data (n = 7), advanced renal impairment (n = 2), and other predefined exclusions (n = 7). This left 70 patients in the eligible, unmatched isolated CAE cohort (Figure 1).

For comparison, 70 patients with angiographically normal coronary arteries (NCA) examined during the same period were identified, resulting in an unmatched cohort of 140 individuals in total. Propensity score matching (PSM) subsequently reduced this to 57 patients per group, for a final matched sample of 114.

### Patient Selection Criteria

Eligibility required that patients be at least 18 years old and show either isolated CAE or angiographically normal coronary arteries. Individuals with conditions likely to alter coronary anatomy, systemic inflammatory status, or lipid profile were excluded—specifically those with acute coronary syndrome, prior coronary artery bypass grafting (CABG), moderate-to-severe valvular disease, malignancy, active infection, chronic inflammatory, or autoimmune conditions, advanced liver disease, severe renal impairment, thyroid dysfunction, corticosteroid use, or incomplete clinical/laboratory records.

Severe renal dysfunction was defined as an estimated glomerular filtration rate (eGFR) below 30 mL/min/1.73 m^2^, while advanced hepatic disease corresponded to Child–Pugh class B or C.

### Angiographic Definitions

A segment was classified as ectatic if its maximal diameter was at least 1.5 times that of a neighboring, angiographically unremarkable reference segment.^3^ For a diagnosis of isolated CAE, at least 1 epicardial artery had to show ectasia in the absence of obstructive CAD, which is defined as ≥50% diameter stenosis in any major epicardial vessel.

Mild-to-moderate stenoses of 1%-49% did not preclude inclusion in the CAE group, but any lesion at or above the 50% threshold led to exclusion from the study. The NCA designation was reserved for participants with entirely unremarkable angiograms—free of stenosis, ectasia, or any other structural abnormality.

All angiograms were read independently by 2 interventional cardiologists, neither of whom had access to clinical or laboratory data. Discordant readings were adjudicated by a third cardiologist, with final group assignment based on consensus.

### Data Collection

Demographic data, cardiovascular risk factors, medication use, anthropometric measurements, and laboratory results from the hospital’s electronic records were extracted. A diagnosis of hypertension, diabetes mellitus, or hyperlipidemia was recorded whenever it had been previously documented or the patient was on disease-specific therapy. Smoking status was coded as positive for both current smokers and those who had quit within the preceding year.

Values for BMI and waist circumference came from routine assessments performed prior to angiography, while laboratory parameters—glucose, triglycerides, total cholesterol, HDL-C, LDL-C, urea, and creatinine—were measured in fasting venous blood drawn before the procedure. Every assay was run in the same institutional laboratory following its standard protocols.

### Visceral Adiposity Index Calculation

Each patient’s VAI was computed with the sex-specific formulas first described by Amato et al,^14^ which combine BMI (kg/m^2^), WCF (cm), TG concentration, and HDL-C. As TG and HDL-C were recorded in mg/dL, both were converted to mmol/L using the standard factors of 88.57 mg/dL per mmol/L for TG and 38.67 mg/dL per mmol/L for HDL-C before applying the equations below:

Female equation:



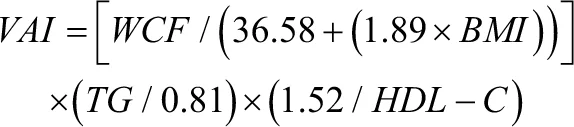



Male equation:




### Statistical Analysis

All statistical analyses were performed with R, version 4.4 (R Foundation for Statistical Computing, Vienna, Austria). Distributional assumptions were assessed visually and using the Shapiro–Wilk test. Continuous variables are presented as mean ± standard deviation for uniform reporting, while the statistical test used for each comparison was selected according to the observed distribution. Categorical variables are presented as counts and percentages.

Group differences in continuous variables were tested with the independent-samples *t*-test when data were normally distributed, or the Mann–Whitney *U*-test otherwise. For categorical variables, the authors used Pearson’s chi-square test, switching to Fisher’s exact test whenever expected cell counts were low.

Propensity scores were derived from a logistic regression model with CAE-versus-NCA status as the dependent variable and age, sex, diabetes mellitus, hypertension, hyperlipidemia, smoking, LDL-C, total cholesterol, systolic blood pressure, and diastolic blood pressure as predictors, all selected in advance for their plausible associations with metabolic risk or coronary remodeling. Variables directly incorporated into the VAI calculation—BMI, waist circumference, triglycerides, and HDL-C—were not included in the propensity score model.

Matching was carried out on a 1 : 1, nearest-neighbor, without-replacement basis, with a caliper width of 0.2 standard deviations of the logit-transformed propensity score. Absolute standardized mean differences were used to assess covariate balance, with values below  0.10 considered acceptable, and the final matched sample retained 57 participants in each group.

In the matched cohort, the relationship between VAI and CAE was examined using logistic regression, beginning with an unadjusted model and an age- and sex-adjusted model. A third model adjusted for the covariates, showing residual post-matching imbalance—diabetes mellitus, smoking status, systolic blood pressure, and diastolic blood pressure. As a post hoc sensitivity analysis, a fourth model that further included total cholesterol was fitted additionally. Results are expressed as odds ratios (OR) with accompanying 95% confidence intervals.

Discriminative performance was evaluated using receiver operating characteristic (ROC) analysis, with the area under the curve (AUC) and its 95% confidence interval estimated. The optimal cut-point was determined by maximizing the Youden index. Sensitivity and specificity were calculated at this optimal threshold. A 2-sided *P* value under .05 was taken to indicate statistical significance throughout.

This study used ChatGPT (OpenAI) to improve language and edit grammar during the preparation of the manuscript. However, all content of the study was reviewed and edited by the authors following the use of artificial intelligence/LLM tools.

## Results

### Findings Before Propensity Score Matching

In the unmatched cohort, no statistically significant differences were observed between the groups in age, sex, diabetes mellitus, hypertension, hyperlipidemia, smoking status, LDL-C, total cholesterol, or systolic and diastolic blood pressure (Table 1; all*P* > .05). Body mass index and waist circumference, however, were both higher in the CAE group (*P* = .007and*P* = .025, respectively), as was mean VAI (2.96 ± 1.51 vs. 2.11 ± 1.15;*P* < .001). Triglycerides were likewise elevated and HDL-C reduced in the CAE group (*P* = .009and *P* < .001, respectively).

### Findings After Propensity Score Matching

The matching process yielded 57 matched pairs. Even after matching, patients with CAE continued to show higher BMI, waist circumference, and triglycerides, along with lower HDL-C, than their NCA counterparts (Table 2; all *P* < .05), and the VAI gap between groups remained substantial (3.01 ± 1.49 vs. 1.95 ± 1.05; *P* < .001).

Post-matching absolute standardized mean differences (SMDs) ranged from 0.000 to 0.147. Residual imbalance remained for diastolic blood pressure (0.147), diabetes mellitus (0.127), systolic blood pressure (0.112), and smoking status (0.109); all other propensity-score covariates had absolute SMDs below 0.10. Full balance statistics are provided in Table 3 and Figure 2. Visceral adiposity index distributions are displayed in Figure 3 and Supplementary Figure 1, while Supplementary Figure 2 summarizes selected metabolic measurements.

### Logistic Regression Analysis

Each 1-unit rise in VAI was associated with 2.21-fold higher odds of CAE in the unadjusted model (95% CI: 1.45-3.36; *P* < .001), an estimate that rose to 2.75 after adjustment for age and sex (95% CI: 1.68-4.48; *P* < .001). After adjustment for the covariates showing residual post-matching imbalance—diabetes mellitus, smoking status, systolic blood pressure, and diastolic blood pressure—the association remained significant (OR: 2.18; 95% CI: 1.43-3.31; *P* < .001). In a post hoc sensitivity model that additionally included total cholesterol, the estimate was similar (OR: 2.24; 95% CI: 1.45-3.45; *P* < .001; Table 4).

### Receiver Operating Characteristics Curve Analysis

Within the matched cohort, VAI yielded an AUC of 0.757 (95% CI: 0.674-0.840). At the Youden-optimal cut-off of approximately 1.97, sensitivity reached 78.9% and specificity 66.7% (Figure 4, Table 5).

## Discussion

The principal finding is that patients with isolated CAE had higher VAI values than angiographically normal controls, a difference that persisted after propensity score matching and remained detectable across multiple regression models. Receiver operating characteristic analysis showed that VAI could distinguish the 2 groups with moderate accuracy. Taken together, these findings suggest a link between the metabolic profile captured by VAI and the ectatic coronary phenotype, consistent with the idea that isolated CAE may reflect more than a purely anatomical abnormality and could be related to a broader cardiometabolic disturbance. Given the retrospective, observational, single-center design of this work, however, the VAI–CAE association cannot be taken as evidence of causation and requires external validation.

Coronary artery ectasia was for a long time viewed simply as an atherosclerotic variant, but a growing body of evidence now points to a more heterogeneous underlying pathophysiology.^4^^,^^6^ Its development is thought to reflect a combination of vessel-wall weakening, aberrant remodeling, chronic inflammation, and extracellular matrix breakdown acting in concert.^6^^,^^7^ As a result, current thinking frames CAE less as a direct consequence of atherosclerotic burden and more as a multifactorial vascular phenotype shaped by inflammatory activation, wall fragility, and dysregulated remodeling.^6^^,^^19^ Viewed against this background, the markedly higher VAI values observed in the CAE group lend support to the notion that visceral-adiposity-related cardiometabolic dysfunction may be associated with this vascular phenotype.

Because it is built from WCF, BMI, TG, and HDL-C, VAI arguably captures more than visceral fat mass alone—it may also register the cardiometabolic changes that accompany adipose tissue dysfunction.^14^^,^^15^ In this sense, VAI goes beyond conventional anthropometry, offering an indirect window onto the biological consequences of dysfunctional fat rather than merely its quantity.^11^^,^^14^ Elevated VAI has been tied, in recent meta-analytic work, to greater risk of cardiovascular disease, coronary artery disease, and cardiovascular death,^13^ yet its specific relationship with CAE has received little dedicated study—a gap these findings help to address by characterizing the visceral-adiposity-related metabolic burden present in isolated CAE. For context, in a large National Health and Nutrition Examination Survey (NHANES)-based cohort, Zhang et al found the highest VAI tertile to be associated with greater odds of self-reported coronary heart disease relative to the lowest tertile (OR: 1.78; 95% CI: 1.32-2.40), while Doganay et al linked VAI to coronary artery calcium score and atherosclerotic plaque morphology.^16^^,^^20^ Neither study used angiographically confirmed CAE or obstructive CAD as its outcome, so their findings are not directly comparable with those of the present study; further research using angiographically defined cohorts is needed to determine whether VAI’s discriminative performance for CAE resembles that reported for other coronary phenotypes.

Two processes are generally regarded as central to CAE pathogenesis: breakdown of the extracellular matrix and abnormal remodeling of the vessel wall.^6^^,^^7^ Heightened matrix metalloproteinase activity, in particular, is thought to degrade elastin and collagen, weakening the vascular wall structurally and setting the stage for ectatic dilation.^21^ A second contributing pathway involves chronic low-grade inflammation—patients with CAE have been reported to show elevated interleukin-6 (IL-6), tumor necrosis factor-alpha (TNF-α), and various adhesion molecules.^2^^,^^22^ Since visceral fat itself functions as an endocrine organ that secretes TNF-α, IL-6, and other adipokines,^15^^,^^23^^,^^24^ excess visceral adiposity could plausibly drive vascular remodeling via endothelial dysfunction, oxidative stress, and proteolytic activity. The observation of elevated VAI in the CAE group is therefore biologically coherent with this proposed mechanism.

There is growing recognition that comparable metabolic and inflammatory disturbances can give rise to quite different cardiovascular phenotypes, reflecting how heterogeneously obesity and visceral adiposity manifest biologically from 1 person to the next.^12^ Obstructive CAD is dominated by plaque formation and luminal narrowing, whereas CAE instead reflects wall weakening and outward dilation—a contrast that raises the possibility of a shared cardiometabolic and inflammatory substrate expressing itself through divergent remodeling pathways. Under this interpretation, VAI is probably better understood not as a direct cause of CAE but as an integrated readout of the underlying metabolic and inflammatory load. The OR for VAI increased after adjustment for age and sex, from 2.21 in the unadjusted model to 2.75. However, because OR from logistic regression models are non-collapsible, this change should not be interpreted as evidence of negative confounding or as indicating a stronger “true” association. In a separate model adjusted for the covariates showing residual post-matching imbalance—diabetes mellitus, smoking status, systolic blood pressure, and diastolic blood pressure—the association remained significant (OR: 2.18; 95% CI: 1.43-3.31;* P* < .001), and a post hoc sensitivity model additionally adjusting for total cholesterol yielded a similar estimate (OR: 2.24; 95% CI: 1.45-3.45; *P* < .001).

That the AUC fell only in the moderate range suggests VAI, while informative about the metabolic phenotype accompanying CAE, has limited value as a stand-alone diagnostic tool. Its comparatively high sensitivity against more modest specificity reinforces the point that VAI should not function as an independent diagnostic test; instead, it is probably best used as a supplementary marker of cardiometabolic risk and visceral-adiposity-related metabolic burden in patients undergoing coronary angiography. Combined with inflammatory biomarkers or imaging-based measures of visceral fat, VAI may add value within a broader, multimarker assessment strategy, pending external validation.

Clinically, the results are consistent with the view that CAE is not merely a localized coronary lesion but a vascular remodeling phenotype associated with systemic cardiometabolic and inflammatory activity—an argument for evaluating the accompanying metabolic risk profile alongside angiographic findings in these patients. Since VAI relies only on routinely collected clinical variables, it could in principle be incorporated into clinical practice, although this would require validation in prospective, multicenter cohorts. These data are not yet sufficient to justify using VAI on its own as a risk-stratification tool.

### Limitations

A number of limitations warrant mention. Being both retrospective and single-center, the study is constrained in its capacity for causal inference and in the generalizability of its conclusions; although PSM improved comparability between groups, residual unmeasured confounding cannot be ruled out. The matched analytical sample was also modest in size—114 patients in total—which likely limits the precision of the estimates and their applicability to other populations. VAI itself, being only an indirect surrogate for visceral fat, cannot substitute for direct imaging-based quantification by computed tomography (CT) or magnetic resonance imaging (MRI), neither of which was available here; nor were inflammatory cytokines, adipokines, or matrix metalloproteinases measured, leaving the mechanistic pathways unverified at the biological level. Finally, variables directly incorporated into the VAI calculation—BMI, waist circumference, triglycerides, and HDL-C—were deliberately excluded from the PSM model to avoid adjustment for components of the exposure itself. Residual differences in these variables persisted between the matched groups and should be considered when interpreting the VAI comparisons reported here. In summary, VAI was higher in patients with isolated coronary artery ectasia than in those with normal coronary arteries, and the association between VAI and CAE remained significant in multivariable analysis—pointing to a possible connection between isolated CAE and the cardiometabolic changes that accompany visceral adiposity. Confirming these observations, and clarifying whether VAI may have a role in metabolic risk assessment for CAE patients, will require prospective, multicenter studies with external validation.

## Supplementary Materials

Supplementary Material

## Figures and Tables

**Figure 1.**
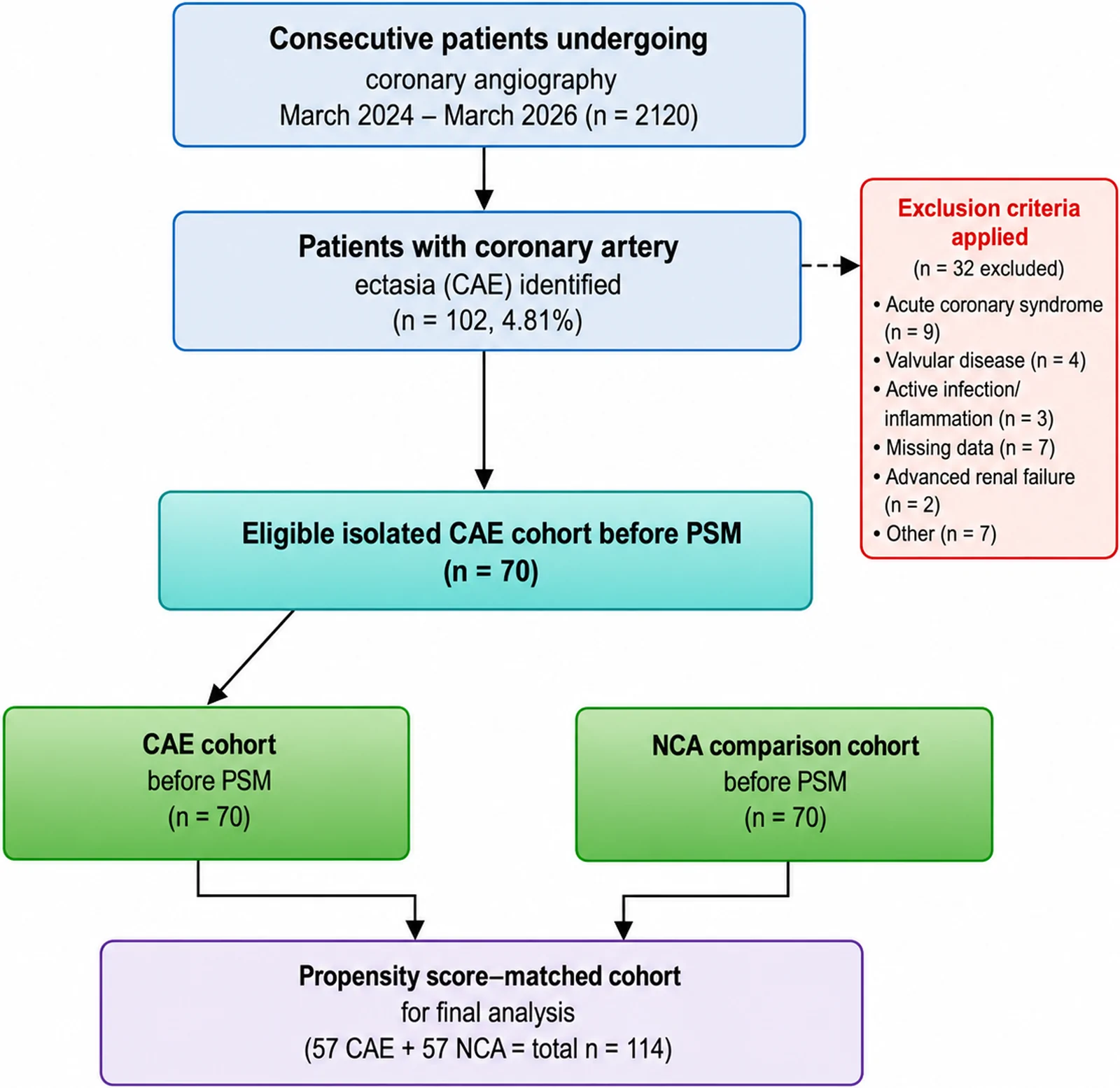
Patient selection and propensity score matching (PSM) flowchart. Illustration of patient identification, application of exclusion criteria, control group selection, and propensity score matching. Of 2120 coronary angiography patients performed from March 1, 2024 to March 1, 2026, coronary artery ectasia (CAE) was detected in 102 patients. After application of the predefined exclusion criteria, 70 patients were eligible for the CAE cohort. Seventy patients with angiographically normal coronary arteries were selected as the initial comparison group. Propensity score matching resulted in a final matched cohort of 57 patients with CAE and 57 patients with normal coronary arteries. CAE, coronary artery ectasia; NCA, normal coronary arteries; PSM, propensity score matching.

**Figure 2.**
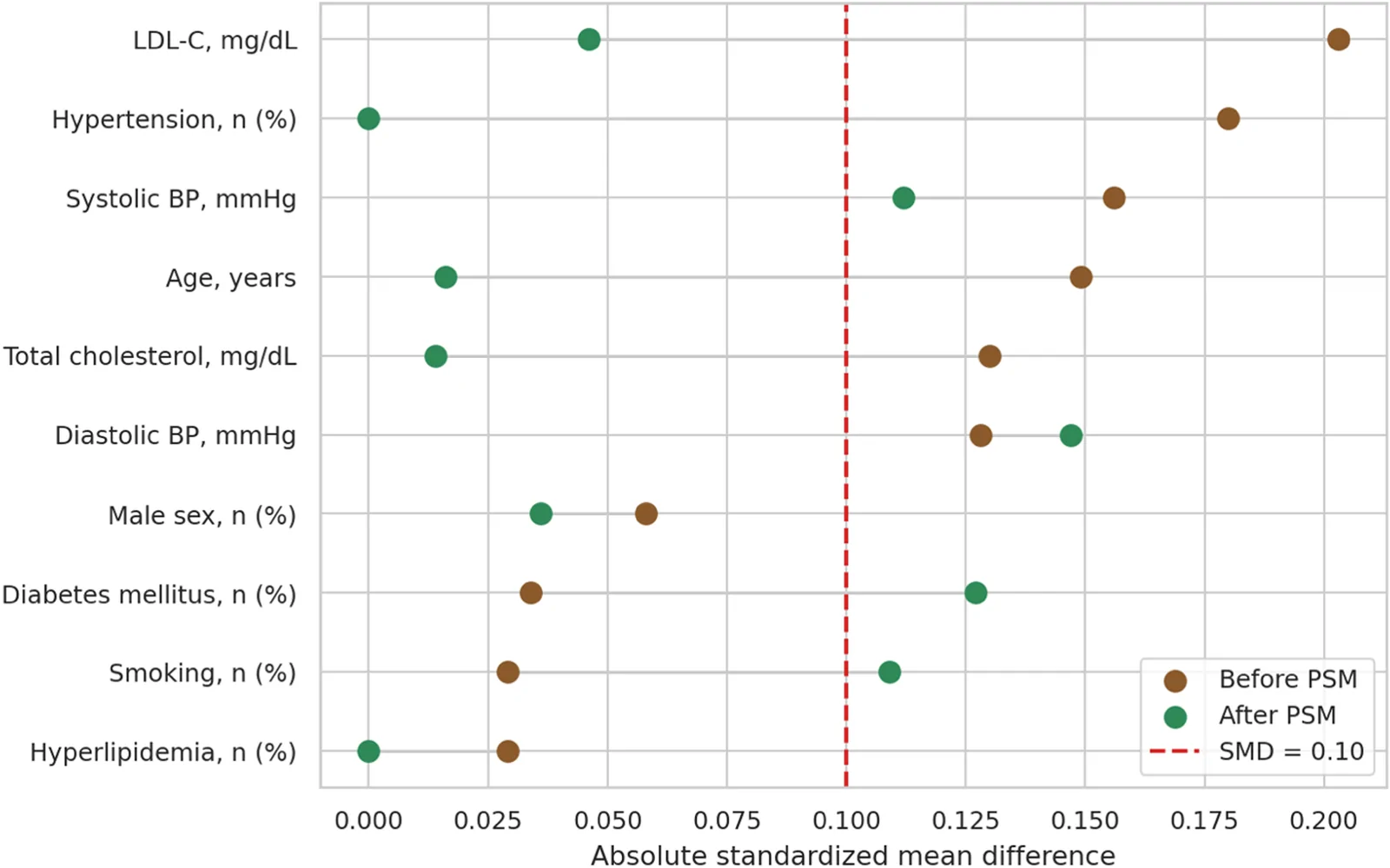
Covariate balance before and after propensity score matching. Absolute standardized mean differences are shown for baseline covariates before and after propensity score matching. The dashed vertical reference line marks the prespecified balance threshold of 0.10. After matching, covariate balance improved substantially, although mild residual imbalance persisted in selected variables. BP, blood pressure; LDL-C, low-density lipoprotein cholesterol; PSM, propensity score matching; SMD, standardized mean difference.

**Figure 3:**
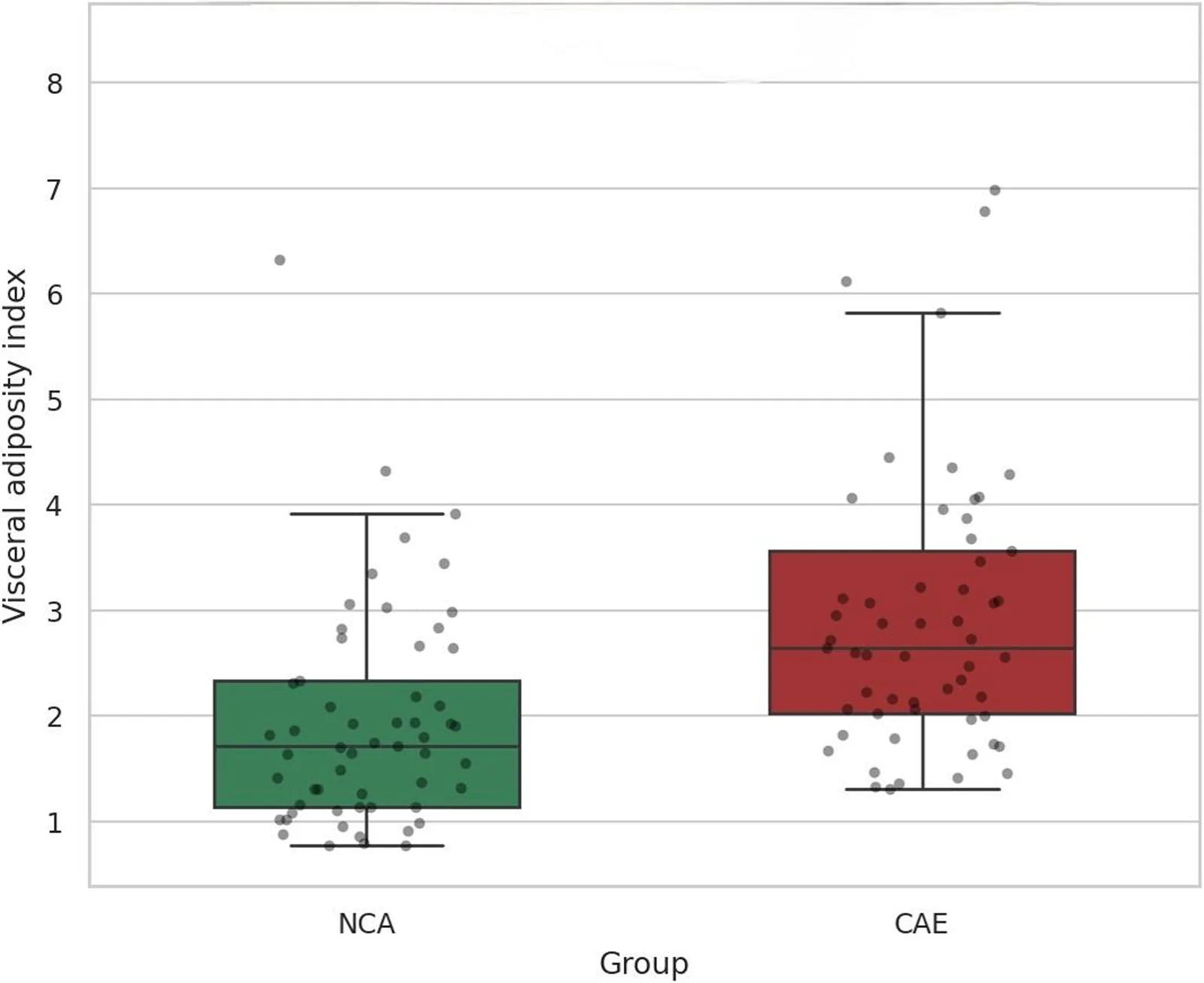
**. **Visceral adiposity index distribution in the matched cohort. Distribution of VAI values in the propensity score–matched cohort according to study group. Patients with CAE had higher VAI values than matched individuals with NCA. CAE, coronary artery ectasia; NCA, normal coronary arteries; VAI, visceral adiposity index.

**Figure 4.**
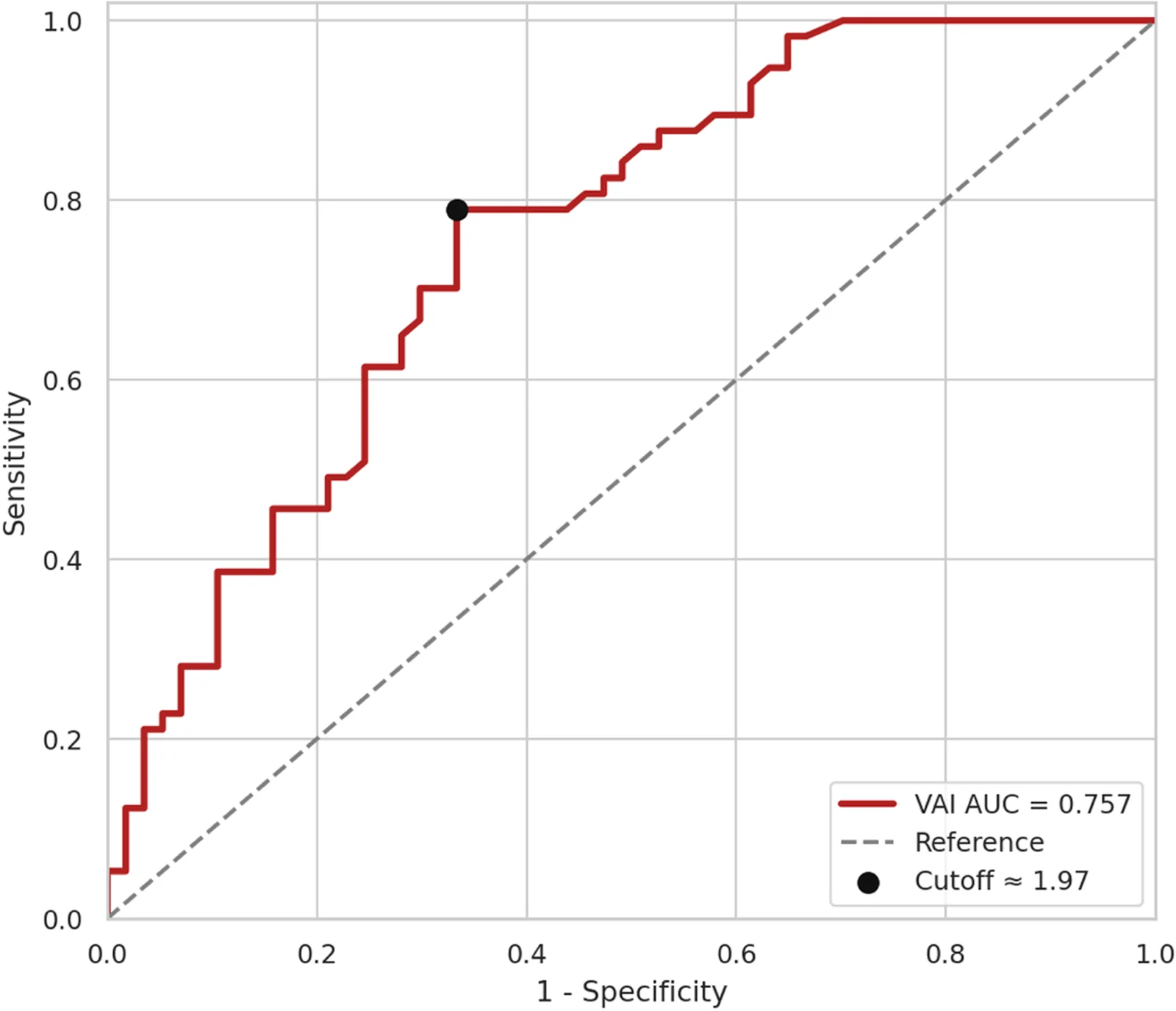
Receiver operating characteristic analysis of VAI for coronary artery ectasia. Receiver operating characteristic curve evaluating the performance of VAI in differentiating CAE from NCA in the matched cohort. VAI demonstrated moderate discriminative ability, with an AUC of 0.757 (95% CI: 0.674-0.840); the Youden index identified 1.97 as the optimal threshold. AUC, area under the curve; CAE, coronary artery ectasia; NCA, normal coronary arteries; ROC, receiver operating characteristic; VAI, visceral adiposity index.

**Table 1.** Pre-Matching Clinical and Laboratory Profile of the Study Groups

Variable	CAE (n = 70)	NCA (n = 70)	*P*
Demographics			
Age, years	56.71 ± 10.47	55.23 ± 9.45	.467
Male sex, n (%)	42 (60.0)	40 (57.1)	.864
BMI, kg/m^2^	28.53 ± 6.13	25.57 ± 3.98	.007
Waist circumference, cm	85.93 ± 16.96	80.21 ± 12.54	.025
Cardiovascular risk factors			
Diabetes mellitus, n (%)	17 (24.3)	16 (22.9)	1.000
Hypertension, n (%)	28 (40.0)	22 (31.4)	.378
Hyperlipidemia, n (%)	29 (41.4)	28 (40.0)	1.000
Smoking, n (%)	27 (38.6)	26 (37.1)	1.000
Adiposity and lipid parameters			
Visceral adiposity index	2.96 ± 1.51	2.11 ± 1.15	<.001
LDL-C, mg/dL	157.60 ± 48.19	147.87 ± 47.55	.231
HDL-C, mg/dL	36.81 ± 9.64	45.41 ± 10.64	<.001
Triglycerides, mg/dL	167.13 ± 55.73	142.87 ± 48.12	.009
Total cholesterol, mg/dL	232.79 ± 50.98	226.42 ± 47.01	.445
Hemodynamic and laboratory parameters			
Systolic BP, mmHg	126.44 ± 19.95	129.29 ± 16.24	.437
Diastolic BP, mmHg	79.69 ± 12.33	81.30 ± 12.83	.480
Hematocrit, %	40.23 ± 5.09	40.04 ± 4.84	.904
WBC, ×10^3^/µL	7.19 ± 2.25	7.34 ± 2.39	.801
Platelet, ×10^3^/µL	286.51 ± 89.29	275.07 ± 78.71	.464
Fasting glucose, mg/dL	115.11 ± 42.92	111.31 ± 40.73	.678
Urea, mg/dL	32.79 ± 7.03	32.50 ± 8.29	.793
Creatinine, mg/dL	0.87 ± 0.23	0.88 ± 0.23	.533

Continuous data are presented as mean ± standard deviation, and categorical data as number (%). Continuous variables were compared using the independent samples *t*-test or Mann–Whitney *U*-test, and categorical variables were compared using the Pearson chi-square test or Fisher exact test, as appropriate.

BMI, body mass index; BP, blood pressure; CAE, coronary artery ectasia; HDL-C, high-density lipoprotein cholesterol; LDL-C, low-density lipoprotein cholesterol; NCA, normal coronary arteries; VAI, visceral adiposity index; WBC, white blood cell count.

**Table 2.** Clinical and Laboratory Profile After Propensity Score Matching

Variable	CAE (n = 57)	NCA (n = 57)	*P*
Demographics			
Age, years	54.79 ± 10.11	54.63 ± 9.55	.984
Male sex, n (%)	35 (61.4)	34 (59.6)	1.000
BMI, kg/m^2^	28.62 ± 5.56	25.45 ± 4.14	.002
Waist circumference, cm	85.70 ± 15.18	79.63 ± 13.08	.028
Cardiovascular risk factors			
Diabetes mellitus, n (%)	11 (19.3)	14 (24.6)	.651
Hypertension, n (%)	20 (35.1)	20 (35.1)	1.000
Hyperlipidemia, n (%)	23 (40.4)	23 (40.4)	1.000
Smoking, n (%)	23 (40.4)	20 (35.1)	.699
Adiposity and lipid parameters			
Visceral adiposity index	3.01 ± 1.49	1.95 ± 1.05	<.001
LDL-C, mg/dL	152.00 ± 49.10	149.75 ± 47.59	.841
HDL-C, mg/dL	36.91 ± 9.47	45.82 ± 9.76	<.001
Triglycerides, mg/dL	173.07 ± 56.00	136.14 ± 42.98	<.001
Total cholesterol, mg/dL	228.47 ± 51.81	227.79 ± 48.48	.980
Hemodynamic and laboratory parameters			
Systolic BP, mmHg	130.72 ± 17.71	128.77 ± 17.09	.628
Diastolic BP, mmHg	82.25 ± 11.31	80.46 ± 13.05	.456
Hematocrit, %	40.21 ± 5.01	39.68 ± 5.06	.639
WBC, ×10^3^/µL	7.27 ± 2.29	7.31 ± 2.37	1.000
Platelet, ×10^3^/µL	282.70 ± 88.67	278.68 ± 76.42	.876
Fasting glucose, mg/dL	111.68 ± 42.16	112.58 ± 44.31	.914
Urea, mg/dL	32.49 ± 6.72	32.09 ± 8.26	.704
Creatinine, mg/dL	0.88 ± 0.23	0.88 ± 0.20	.752

Data are reported as mean ± standard deviation or number (%), depending on variable type. Variables directly incorporated into the VAI calculation—BMI, waist circumference, triglycerides, and HDL-C—were not included in the propensity score model. Therefore, residual differences in these variables should be considered when interpreting post-matching comparisons.

BMI, body mass index; BP, blood pressure; CAE, coronary artery ectasia; HDL-C, high-density lipoprotein cholesterol; LDL-C, low-density lipoprotein cholesterol; NCA, normal coronary arteries; PSM, propensity score matching; VAI, visceral adiposity index; WBC, white blood cell count.

**Table 3.** Covariate Balance Before and After Propensity Score Matching

Covariate	|SMD| before PSM	|SMD| after PSM
Age, years	0.149	0.016
Male sex	0.058	0.036
Diabetes mellitus	0.034	0.127
Hypertension	0.180	0.000
Hyperlipidemia	0.029	0.000
Smoking	0.029	0.109
LDL-C, mg/dL	0.203	0.046
Total cholesterol, mg/dL	0.130	0.014
Systolic BP, mmHg	0.156	0.112
Diastolic BP, mmHg	0.128	0.147

Covariate balance was evaluated using absolute standardized mean differences. An absolute SMD below 0.10 was prespecified as the criterion for satisfactory covariate balance. After matching, overall covariate balance improved; however, residual imbalance remained for diabetes mellitus, smoking status, systolic blood pressure, and diastolic blood pressure.

BP, blood pressure; LDL-C, low-density lipoprotein cholesterol; PSM, propensity score matching; SMD, standardized mean difference.

**Table 4.** Logistic Regression Models for the Association Between VAI and CAE in the Propensity Score–Matched Cohort

Model	Adjustment Variables	OR per 1-unit increase in VAI	95% CI	*P*
Model 1	None	2.21	1.45-3.36	<.001
Model 2	Age, sex	2.75	1.68-4.48	<.001
Model 3	Diabetes mellitus, smoking status, systolic BP, diastolic BP	2.18	1.43-3.31	<.001
Model 4 (sensitivity)	Diabetes mellitus, smoking status, total cholesterol, systolic BP, diastolic BP	2.24	1.45-3.45	<.001

Logistic regression models were fitted in the propensity score–matched cohort. Model 1 represents the unadjusted estimate. Model 2 was adjusted for age and sex. Model 3 was adjusted for diabetes mellitus, smoking status, systolic blood pressure, and diastolic blood pressure. Model 4 was a post hoc sensitivity model that additionally included total cholesterol. Results are presented as odds ratios with 95% confidence intervals.

BP, blood pressure; CAE, coronary artery ectasia; DBP, diastolic blood pressure; NCA, normal coronary arteries; OR, odds ratio; SBP, systolic blood pressure; VAI, visceral adiposity index.

**Table 5.** ROC-Derived Performance of VAI for Discriminating CAE from NCA

Analysis	AUC (95% CI)	Sensitivity	Specificity	Optimal Cutoff
PSM cohort (n = 114)	0.757 (0.674-0.840)	78.9%	66.7%	≈1.97

The ability of VAI to distinguish patients with CAE from those with NCA was assessed using receiver operating characteristic curve analysis in the propensity score–matched cohort. The threshold maximizing the sum of sensitivity and specificity was identified via the Youden index.

AUC, area under the curve; CAE, coronary artery ectasia; NCA, normal coronary arteries; PSM, propensity score matching; ROC, receiver operating characteristic; VAI, visceral adiposity index.

## Data Availability

The data that support the findings of this study are available on request from the corresponding author.
