# Supplementary material for: Visceral Adiposity Index in Coronary Artery Ectasia
Source: Eurasian J Med. 2026 Sep 23;58(4):e261588. doi: 10.5152/eurasianjmed.2026.261588 (PMC13643854; doi:10.5152/eurasianjmed.2026.261588)
Supplement: Supplementary file 1 [file supplementary_material.pdf]

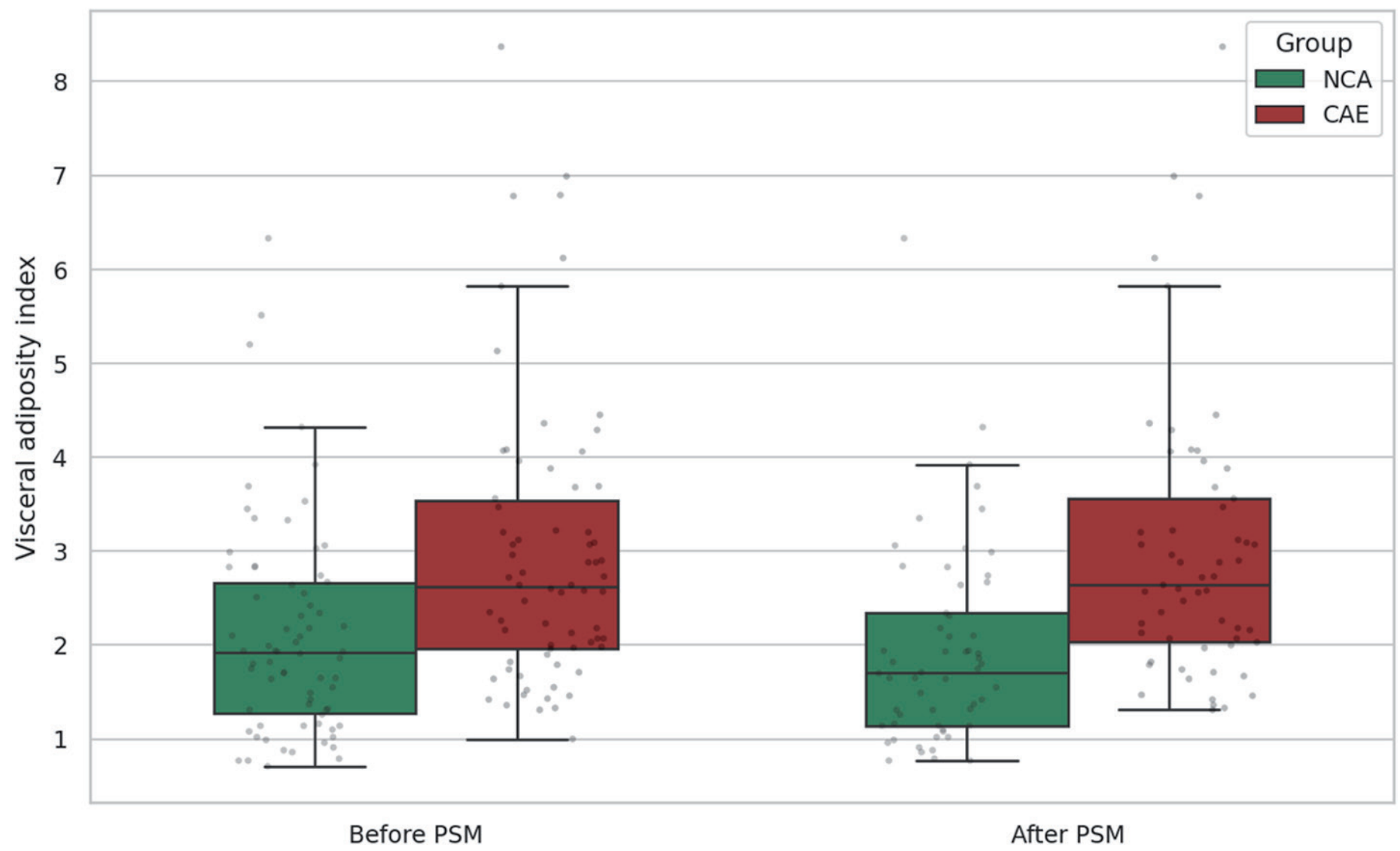

Supplementary Figure 1. VAI distribution before and after propensity score matching. VAI distributions are presented for the CAE and NCA groups in both the unmatched and propensity score–matched cohorts. Higher VAI values in the CAE group were observed before and after matching. Abbreviations: CAE, coronary artery ectasia; NCA, normal coronary arteries; PSM, propensity score matching; VAI, visceral adiposity index.

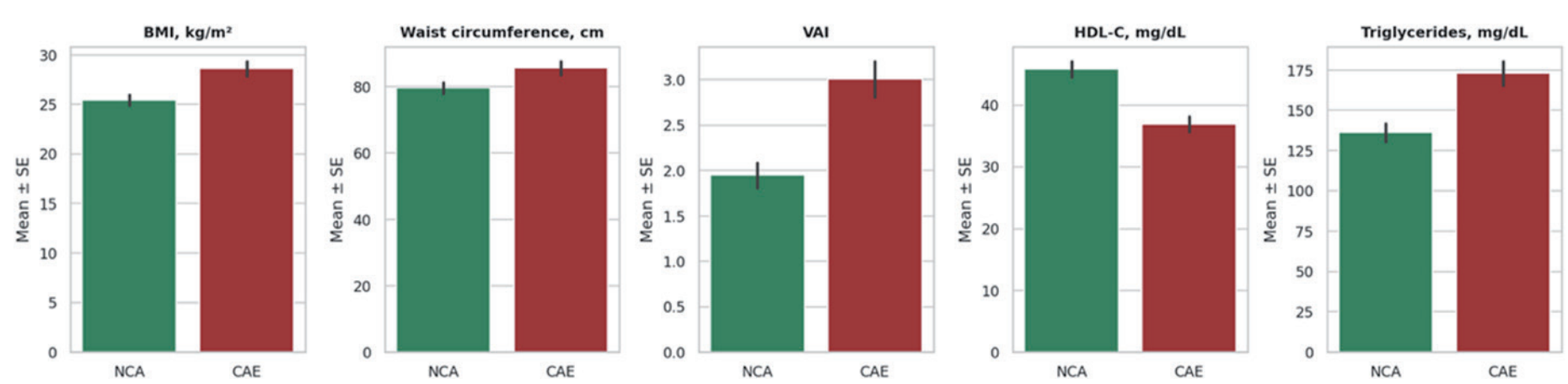

Supplementary Figure 2. Metabolic and adiposity-related variables in the matched cohort. Bar plots summarize selected variables after propensity score matching, including BMI, waist circumference, VAI, HDL-C, and TG. Error bars indicate the standard error of the mean. Abbreviations: BMI, body mass index; HDL-C, high-density lipoprotein cholesterol; TG, triglycerides; VAI, visceral adiposity index.
